# Prognostic value of the MicroRNA regulators Dicer and Drosha in non-small-cell lung cancer: co-expression of Drosha and miR-126 predicts poor survival

**DOI:** 10.1186/1472-6890-14-45

**Published:** 2014-12-11

**Authors:** Kenneth Lønvik, Sveinung W Sørbye, Marit N Nilsen, Ruth H Paulssen

**Affiliations:** Department of Clinical Pathology, University Hospital of Northern Norway, N-9038 Tromsø, Norway; Department of Medical Biology, Tromsø, Norway; Department of Clinical Medicine, UiT – The Arctic University of Norway, N-9037 Tromsø, Norway

**Keywords:** NSCLC, Dicer, Drosha, microRNA, miR-126, Immunohistochemistry

## Abstract

**Background:**

Dicer and Drosha are important enzymes for processing microRNAs. Recent studies have exhibited possible links between expression of different miRNAs, levels of miRNA processing enzymes, and cancer prognosis. We have investigated the prognostic impact of Dicer and Drosha and their correlation with miR-126 expression in a large cohort of non-small cell lung cancer (NSCLC) patients. We aimed to find patient groups within the cohort that might have an advantage of receiving adjunctive therapies.

**Methods:**

Dicer expression in the cytoplasm and Drosha expression in the nucleus were evaluated by manual immunohistochemistry of tissue microarrays (TMAs), including tumor tissue samples from 335 patients with resected stages I to IIIA NSCLC. In addition, *in situ* hybridizations of TMAs for visualization of miR-126 were performed. Kaplan–Meier analysis was performed, and the log-rank test via SPSS v.22 was used for estimating significance levels.

**Results:**

In patients with normal performance status (ECOG = 0, n = 197), high Dicer expression entailed a significantly better prognosis than low Dicer expression (P = 0.024). Dicer had no significant prognostic value in patients with reduced performance status (ECOG = 1–2, n = 138). High Drosha expression was significantly correlated with high levels of the microRNA 126 (miR-126) (P = 0.004). Drosha/miR-126 co-expression had a significant negative impact on the disease-specific survival (DSS) rate (P < 0.001). Multivariate analyses revealed that the interaction Dicer*Histology (P = 0.049) and Drosha/miR-126 co-expression (P = 0.033) were independent prognostic factors.

**Conclusions:**

In NSCLC patients with normal performance status, Dicer is a positive prognostic factor. The importance of Drosha as a prognostic factor in our material seems to be related to miR-126 and possibly other microRNAs.

**Electronic supplementary material:**

The online version of this article (doi:10.1186/1472-6890-14-45) contains supplementary material, which is available to authorized users.

## Background

Lung cancer is a heterogeneous disease and a leading cause of cancer-related death in most developed countries. Although there have been advances in treatment over the past few years, the 5-year disease-specific survival (DSS) rate is still < 15%. Therefore, it is important to investigate possible prognostic factors among the survivors in order to gain a better understanding of NSCLC malignancy and to develop treatment options for different NSCLC patient subgroups [1].

Recently, an increasing number of reports have implicated a role for miRNAs in lung cancer progression [2, 3]. MicroRNAs are potential targets for treating NSCLC carcinomas [4], and research has focused on the diagnostic and prognostic potential of different microRNAs (miRNAs or miRs) in NSCLC. It is believed that miRNA expression is important in NSCLC development [5, 6]. Expression profiling of miRNAs in normal and diseased lung tissues have revealed unique expression patterns, and a number of miRNAs have been characterized as tumor suppressor genes or oncogenes [7–13].

Several studies have identified miR-126 as a novel prognostic marker for predicting the overall survival rate of patients with some types of cancer [14, 15]. MiR-126 has been found to be expressed predominantly by endothelial cells, thereby influencing angiogenesis [16, 17] by downregulation of VEGF-A expression through the interaction with the 3’-untranslated region [18]. An independent and tissue-specific prognostic impact of miR-126 has been demonstrated in NSCLC, where co-expression of miR-126 with vascular endothelial growth factor-A (VEGF-A) predicts poor survival [19]. Other research has implied that mir-126 inhibits tumor cell growth, and its expression level correlates with poor survival of NSCLC patients [7]. The expression and roles of miR-126 might be different in various malignancies where miR-126 is downregulated, thereby acting as potential tumor suppressor [19–22].

Understanding the biogenesis of miRNAs has caught the interest of many researchers, and several papers have been published that focus on the enzymes necessary for synthesizing miRNAs [23–25]. MicroRNAs are generated in a two-step processing pathway mediated by two major enzymes, Dicer and Drosha, both of which belong to the class of RNase III endonucleases. The intranuclear miRNA processing enzyme Drosha and the extranuclear microRNA-processing enzyme Dicer play pivotal roles in miRNA maturation. Drosha is part of a multiprotein complex that mediates the nuclear processing of the primary miRNAs into stem-loop precursors (pre-miRNA). In the cytoplasm, the pre-miRNA is cleaved by Dicer into mature nucleotide miRNA. In the biogenesis of the majority of miRNAs, both Dicer and Drosha are necessary factors, together with several other proteins involved in the miRNA processing machinery [23, 26]. Dicer and Drosha seem to have a prognostic impact, and both have been found to be differentially expressed in various cancer tissue types when compared to normal tissue [27–32].

The Eastern Cooperative Oncology Group (ECOG) performance status [33] provides scales and criteria for assessing how a patient’s disease is progressing and helps to determine appropriate treatment options and prognosis. Many studies include patients with an ECOG performance status grade of 0 and 1 only. Patients in these groups are either, fully active and able to carry on all pre-disease performance without restrictions, or are restricted in physically strenuous activity and able to carry out work of a light or sedentary nature. In this study we have also included NSCLC patients with an ECOG grade of 0–2 that are capable of all self-care but unable to carry out any work activities and have more than 50% of waking hours. Cancer patients with an ECOG grade of 3–4 have reduced survival regardless of other clinical and pathological variables.

Although several studies have been performed on different cancer types in order to elucidate and decipher the roles of Dicer and Drosha in carcinogenesis and their potential impact on prognosis, the contribution of Dicer and Drosha on miR-126 expression in NSCLC has not been addressed. Therefore, this study investigates the possible prognostic value of the expression of the miRNA regulators Dicer and Drosha on miR-126 processing in a NSCLC patient cohort.

## Methods

### Ethics statement

The study was approved by The National Data Inspection Board, The Regional Committee for Research Ethics (REK Nord). The Regional Committee for Research Ethics specifically waived the need for consent, since this is a retrospective study with more than half of patients deceased.

### Patients and clinical samples

The study examined primary tumor tissues from anonymized patients diagnosed with NSCLC pathologic stage I to IIIA within the period from 1990 to 2004 at the University Hospital of North Norway (UNN) and Nordland Central Hospital (NLCH). During this period adjuvant chemotherapy had not yet been introduced in Norway. Thus, 371 patients were considered as potential candidates for this study, of which 36 patients were excluded due to (i) chemotherapy or radiotherapy prior to surgery (n = 10), (ii) other malignancy within five years prior to NSCLC diagnosis (n = 13), and (iii) inadequate paraffin-embedded fixed tissues (n = 13). The analysis was, therefore, left with 335 patients with complete medical records and adequate paraffin-embedded tissues. All prognostic clinicopathologic variables as predictors for DSS in 335 NSCLC patients are summarized in Additional file 1: Table S1 and were reported in a previous study [19].

The NSCLC patients included in this study have an ECOG rating of 0, 1, or 2, where normal performance status is equal to 0 and reduced performance status is 1 or 2. NSCLC patients are rated from 0–5, but only patients with ratings from 0–2 are eligible for surgery. The rating system has been explained in detail in previous publications [33] and http://www.ecog.org/general/perf_stat.html.

All tumor tissues were selected at primary surgery of previous non-treated lung cancer patients and the follow-up started directly after surgery. The last follow-up data included was from November 30, 2008. The median follow-up for survivors was 86 (range 48–216) months. The tumors were staged according to the new 7th edition of TNM classification in Lung Cancer and histologically subtyped and graded according to the World Health Organization’s guidelines [34, 35].

### Immunohistochemistry (IHC)

All lung cancer cases were histologically reviewed by two experienced pathologists (Samer Al-Saad and Khalid Al-Shibli), and the most representative areas of viable tumor cells (neoplastic epithelial cells) were carefully selected. Within these areas, four cores from each patient were randomly sampled and assembled in TMA (tissue microarray) blocks. The detailed methodology has been reported previously [36]. As controls served samples of normal lung tissue localized distant from the primary tumor, and normal lung tissue samples from 20 patients without any cancer diagnosis (see Additional file 2: Figure S1). Multiple 4 μm sections were cut with a Microm microtome (HM355S) and analyzed via immunohistochemistry with regard to the miRNA regulators Dicer and Drosha.

Specific antibodies for Dicer (13D6-ChIP grade, ab14601) and Drosha (ab85027) (both Abcam, Cambridge, UK) have been validated in-house by the manufacturer for IHC analysis on paraffin-embedded material prior to use. Sections were deparaffinized with xylene and rehydrated through graded ethanol series (Drosha) or by using Ventana reagents for automatic staining of Dicer (Ventana BenchMark XT, Ventana Medical Systems Inc.). Manual antigen retrieval (for Drosha) was performed by placing the specimens in a 10 mM Tris–HCl/1 mM EDTA buffer, with pH 9.0, and subsequent microwave heating for 20 minutes at 450 W. Automatic antigen retrieval (for Dicer) (Ventana Benchmark XT) was performed by heating the sections for 30 minutes in a Tris-based buffer (CC1 mild). Staining was performed with a detection reagent containing a secondary antibody plus an avidin-biotin enzyme complex (manual procedure), or a polymer of secondary antibodies conjugated with an enzyme (Ventana). Primary antibodies were diluted at 1:20 (Dicer) and 1:100 (Drosha) or incubated overnight at room temperature (Drosha) and for 32 minutes at 37°C (Dicer). Diaminobenzidine (DAB) was used to visualize the antigens. The detection system in the Ventana XT was the ultraView DAB. Finally, counterstaining was performed with hematoxylin and by mounting the slides. In negative control slides, the primary antibody was replaced with the primary antibody diluent, and for positive staining controls, we used breast carcinoma samples (data not shown).

### *In Situ*Hybridization (ISH)

The *in situ* hybridization method was adapted from [37] and performed with minor adjustments due to different batches of labelled probes. *In situ* hybridizations of TMA sections for visualization of miR-126 were essentially performed in accordance with recent research [19].

### Scoring of IHC

**Figure 1:**
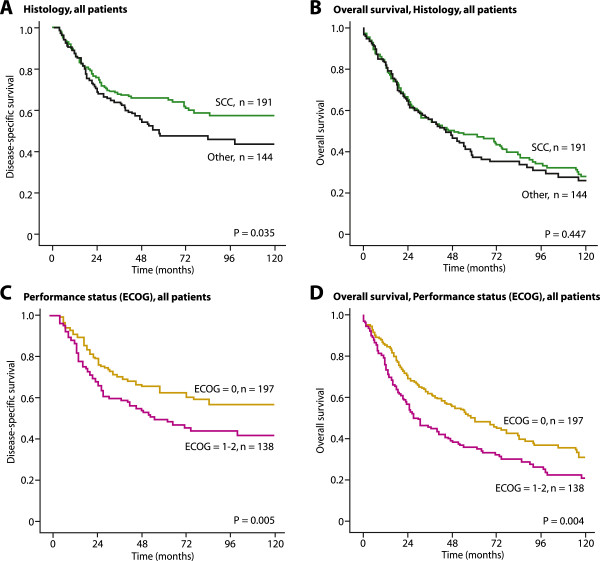
**Disease-specific survival and overall survival curves for histology (A and B) and ECOG (C and D) including all patients.** SCC indicates squamous cell carcinoma.

**Figure 2:**
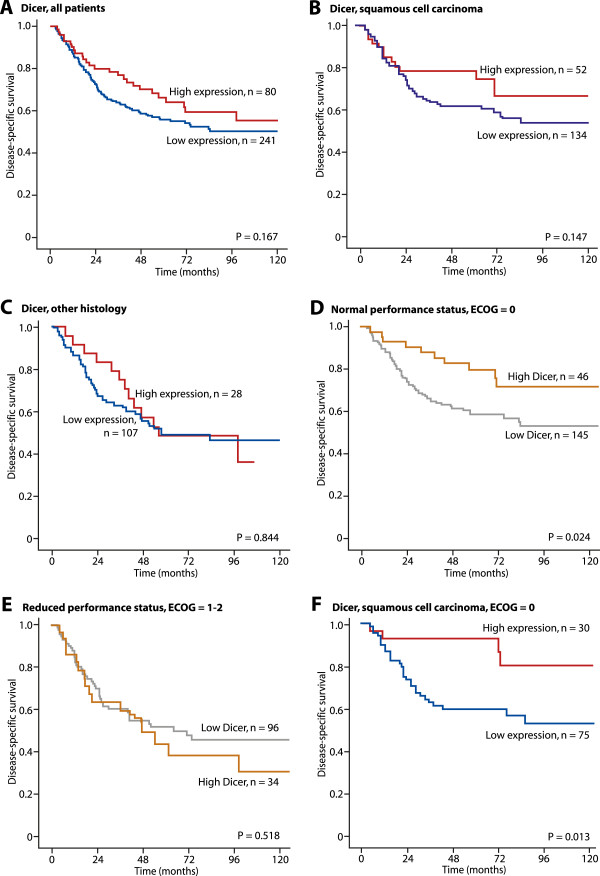
**Disease-specific survival curves for high and low expression of Dicer in NSCLC patients (n = 321) (A), in patients with squamous cell carcinoma (n = 186) (B), in patients with other histology (n = 135) (C), in patients with normal performance status (ECOG = 0, n = 191) (D), in patients with reduced performance status (ECOG = 1–2, n =140) (E), and in patients with squamous cell carcinoma with normal performance status (ECOG = 0, n = 105) (F).**

The IHC-stained TMA slides were scanned with the ARIOL imaging system (Genetix, San Jose, CA) as follows: The slides were loaded in the automated loader (Applied Imaging SL 50) and TMA slides were scanned at low (1.25 x) and high resolutions (20 x) by using the Olympus BX 61 microscope with an automated platform. Representative and viable tissue sections were scored manually and semi-quantitatively for cytoplasmic staining (Dicer) and for staining the tumor cell nuclei (Drosha) via a computer screen. The average staining intensity of the majority of cells was scored as 0 = negative, 1 = weak, 2 = intermediate, and 3 = strong (see Figures 1 and 2), as described previously [36]. In case of disagreement (score variance > 1), the slides were re-examined and an agreement was reached by the observers. In most cores there was a mixture of stromal cells and tumor cells. By morphological criteria only tumor cells were scored for staining intensity.

All samples were anonymized and independently scored by an experienced pathologist and a technician (S.W.S. and K.L.). When scoring the samples, the observers were blind to the scores of the other observer and to the outcome. The mean score for each case was calculated from all four cores by both examiners. High expression of both Dicer and Drosha in neoplastic tumor cells was defined as a mean score ≥ 2. This cut-off value was selected to find the two groups with the largest possible difference in survival. It is hereby noted that the results might be depended on the choice of the cut-off value. However, for miR-126 we used the same cut-off value and the same scoring system as previously described in detail [19, 38].

### Inter-observer variability

An inter-observer scoring agreement was tested for both Dicer and Drosha, and the agreement was robust (r = 0.92, P < 0.001).

### Statistical methods

In brief, statistical analyses were conducted with the statistical package SPSS (Chicago, IL), version 22. The Chi-square test and Fisher’s exact test were used to examine the association between Dicer and Drosha expressions and various clinicopathological parameters. The IHC scores from each observer were compared for inter-observer reliability by use of a two-way random effect model with absolute agreement definition. The intraclass correlation coefficient (reliability coefficient) was obtained from these results. The Kaplan–Meier method was used to plot DSS according to expression levels, and statistical significance between survival curves was assessed by the log-rank test. DSS was determined from the date of surgery to the time of death from lung cancer. The multivariate analysis was conducted with the Cox proportional hazards model. Only those variables of significant value from the univariate analysis were entered into the Cox regression analysis. Differences in expression of Dicer, Drosha, and miR-126 as continuous variables by histology are analyzed using ANOVA. The significance level employed was P < 0.05.

## Results

### Overall NSCLC patient group characteristics

The NSCLC patient cohort comprised 253 males (75%) and 82 females (25%), (Additional file 1: Table S1). The follow-up time was up to 250 months (20 years). During follow-up, 236 (70%) patients died, 137 (40%) from lung cancer and 99 (30%) from other reasons (data not shown). The 5- year DSS was 56% for males and 63% for females (Additional file 1: Table S1). On a continuous scale (0–3), the mean expression of Dicer, Drosha, and miR-126 in all patients was 1.18, 1.41, and 1.14, respectively. Patients with squamous cell carcinoma had significantly higher expression of Dicer and miR-126 than patients with adenocarcinoma (Additional file 1: Table S2). Using ≤ 2.0 as a cut-off of expression (Additional file 2: Figure S1), about 70–80% of the patients had low expression and 20–30% of the patients had high expression of Dicer, Drosha, and miR-126 (data not shown).

### Performance status (ECOG), disease-specific survival (DSS), and overall survival (OS) in NSCLC patient groups—Univariate analyses

WHO performance status (ECOG, P = 0.013), histology (P = 0.028), tumor differentiation (P < 0.001), surgical procedure (P < 0.004), pathological stage (P < 0.001), tumor status (P < 0.001), nodal status (P < 0.001), and vascular infiltration (P < 0.001) were all significant indicators for DSS in univariate analyses (Additional file 1: Table S1 and [19]).

Patients with squamous cell carcinoma had significantly longer DSS than lung cancer patients with other histology (P = 0.035, Figure 1A). However, there were no significant differences regarding overall survival between patients with squamous cell carcinoma compared to patients with other histology (P = 0.447, Figure 1B). Patients with normal performance status (ECOG = 0) had significantly longer DSS and overall survival than patients with reduced performance status (ECOG = 1–2), P = 0.005 (Figure 1C) and P = 0.004 (Figure 1D).

### Dicer and Drosha expressions and correlations

Dicer was expressed in the cytoplasm of most neoplastic tumor cells, and slight staining was observed in the nucleus of some cells as well (Additional file 2: Figure S2). Thirty-five patients (10.4%) scored negative on all four cores. Drosha was primarily expressed in the nucleus, but some staining was observed in the cytoplasm (Additional file 2: Figure S3). We found 14 patients (4.2%) that scored negative on all four sample cores. Inflammatory cells, pneumocytes, and fibroblasts showed greater variability in expressing both Dicer and Drosha when compared to tumor cells. A significant, but low correlation between Dicer expression in cytoplasm and Drosha expression in the nucleus of neoplastic tumor cells (r = 0.473, P < 0.001) was observed (Additional file 2: Figure S4).

**Figure 3:**
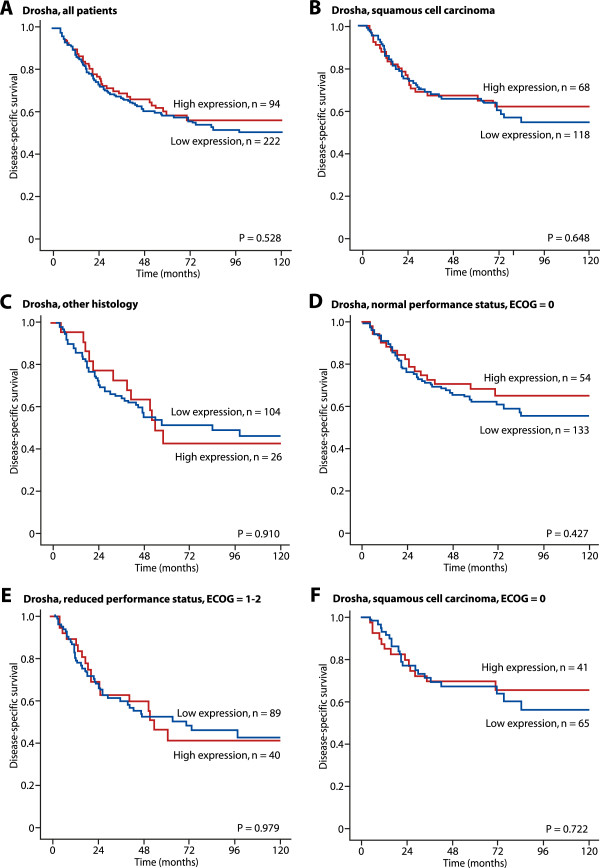
**Disease-specific survival curves for high and low expression of Drosha in NSCLC patients (n = 316) (A), in patients with squamous cell carcinoma (n = 186) (B), in patients with other histology (n = 130) (C), in patients with normal performance status (ECOG = 0, n = 187) (D), in patients with reduced performance status (ECOG = 1–2, n =129) (E), and in patients with squamous cell carcinoma with normal performance status (ECOG = 0, n = 116) (F).**

The expression of Dicer (P = 0.008) and miR-126 (P = 0.020) was different in the tumor subgroups (see Additional file 1: Table S2). Expression of Dicer was not correlated with DSS when all patients were analyzed in one group (P = 0.167, Figure 2A). The expression of Dicer was of not correlated with DSS in patients with squamous cell carcinoma (P = 0.147, Figure 2B) or other histologies (P = 0,844, Figure 2C). However, in patients with normal performance status (ECOG = 0), high expression of Dicer was significantly correlated with longer DSS (P = 0.024,) (Figure 2D), but no differences in patients with reduced performance status (ECOG = 1–2) were observed (P = 0.518, Figure 2E). In patients with both squamous cell carcinoma and normal performance status (ECOG = 0), high expression of Dicer was significantly correlated with long DSS (P = 0.013, Figure 2F). Expression of Drosha was not correlated with DSS for any subgroups of histology and performance status (Figure 3A– F).

**Figure 4:**
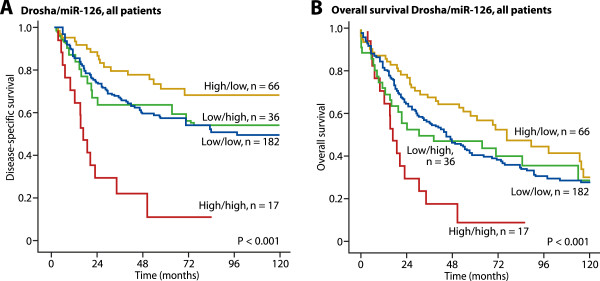
**Disease-specific survival (A) and overall survival (B) curves for co-expression of Drosha and miR-126 in the total patient material (n = 301).**

When combining expression of Drosha and miR-126, the subgroup of patients with both high Drosha and high miR-126 expression had significantly shorter DSS (P < 0.001) (Figure 4A) and overall survival (P = 0.001, Figure 4B).

### Multivariate cox proportional hazard analysis

Tumor status (T-stage, P = 0.002), nodal status (N-stage, P < 0.001), performance status (0.003), vascular infiltration (P = 0.016), interaction of Dicer with histology (Dicer*Histology, P = 0.049), and Drosha/miR-126 co-expression (P = 0.033) were independently significantly correlated with DSS (Table 1). In the subgroup of patients with both squamous cell carcinoma and normal performance status (ECOG = 0, n = 107), tumor status (P = 0.021), nodal status (P = 0.001), vascular infiltration (P = 0.011), and expression of Dicer (P = 0.031), but not tumor differentiation (P = 0.587), were independently correlated with DSS (Table 2).

**Table 1:** **Cox regression analysis summarizing significant independent prognostic factors**

Factor	HR	95% CI	P
**Tumor status**			0.002*
1	1.00		
2	1.54	0.91 – 2.59	0.108
3	2.73	1.53 – 4.90	0.001
**Nodal status**			<0.001*
0	1.00		
1	1.98	1.27 – 3.08	0.003
2	3.13	1.80 – 5.46	<0.001
**Histology**			
SCC	1.00		
Other NSCLC	0.99	0.500 – 1.98	0.986
**Differentiation**			0.094*
Poor	1.00		
Moderate	0.70	0.46 – 1.05	0.083
Well	0.56	0.29 – 1.10	0.095
**Performance status**			
ECOG = 0	1.00		
ECOG = 1-2	1.80	1.23 – 2.64	0.003
**Vascular infiltration**			
No	1.00		
Yes	1.82	1.12 – 2.98	0.016
Dicer**	0.87	0.65 – 1.16	0.333
Dicer*Histology***	1.27	1.00 – 1.61	0.049
**Drosha / miR-126**			0.033*
Low / Low	1.00		
Low / High	1.13	0.62 – 2.05	0.696
High / Low	0.63	0.37 – 1.07	0.089
High / High	1.96	1.04 – 3.69	0.037

*Overall significance as a prognostic factor.

**B = −0.141.

***B = 0.240.

Dicer*Histology = the interaction between Dicer and Histology.

HR: Hazard ration; CI: confidence interval.

**Table 2:** **Cox regression analysis summarizing significant independent prognostic factors in patients with squamous cell carcinoma and normal performance status (ECOG = 0), n = 107**

Factor	HR	95% CI	P
**Tumor status**			0.021*
1	1.00		
2	2.52	0.82 – 7.79	0.108
3	5.11	1.54 – 16.89	0.008
**Nodal status**			0.001*
0	1.00		
1	2.11	1.21 – 2.89	0.045
2	20.06	3.70 – >99	0.001
**Differentiation**			0.587*
Poor	1.00		
Moderate	0.64	0.26 – 1.56	0.328
Well	0.72	0.23 – 2.26	0.578
**Vascular infiltration**			
No	1.00		
Yes	3.10	1.29 – 7.42	0.011
**Dicer**			
Low	1.00		
High	0.30	0.10 – 0.90	0.031

*Overall significance as a prognostic factor.

HR: Hazard ratio; CI: confidence interval.

When Dicer and Drosha are analyzed on a continuous scale (0–3) instead of two groups of expression (high and low), none of them correlated with DSS in the total patient material (Additional file 1: Table S3). To identify other relevant factors regarding expression of Dicer, the expression was tested by histology, performance status, tumor differentiation, tumor status, nodal status, and vascular infiltration. High expression was found in SCC (P = 0.012) and in tumors with vascular infiltration (P = 0.035, Additional file 1: Table S4). The Dicer interaction with histology was highly significant (P = 0.005, Additional file 1: Table S5). This was not the case for Dicer interaction with ECOG (Additional file 1: Table S6) and Dicer interaction with vascular infiltration (Additional file 1: Table S7). The proportionality of hazards was tested graphically and the results are depicted in Additional file 2: Figure S5.

### Co-expression of Drosha and miR-126

For co-expression analyses, we used miR-126 data obtained by ISH of TMAs from a previously published study [19] demonstrating that high miR-126 expression is an independent negative prognostic factor in the total patient cohort. As illustrated in Figure 4, a univariate analysis found that the co-expression of Drosha and miR-126 had a significant impact on DSS, with 5-year survival rates of 58% (patients with low Drosha and low miR-126 expression), 61% (low Drosha and high miR-126 expression), 74% (high Drosha and low miR-126 expression), and 17% (high Drosha and high miR-126 expression) (p < 0.001). In a multivariate analysis, these co-expressions were independent prognostic indicators for DSS (p = 0.016). For patients with high Drosha/high miR-126, the HR was 2.1 (1.1–4.0 at 95% CI, p < 0.001) compared to patients with high Drosha/low miR-126 (Table 1).

## Discussion

In this large-scale study comprising primary tumors from 335 patients, we investigated the prognostic impact of the miRNA regulators Dicer and Drosha in NSCLC. The observed expression of the nuclear enzyme Drosha and the cytoplasmic enzyme Dicer correlated positively with each other, suggesting their mutual dependence in the miRNA-regulatory pathway in NSCLC. Reduced Dicer and Drosha expression has been reported in various cancers, and based on previous publications on NSCLC [28, 29], there was an expectation that both Dicer and Drosha would have a positive prognostic impact on DSS, where reduced Dicer and Drosha expression would entail a poorer prognosis compared to higher expressions. Our results found no significant association between the miRNA regulators and DSS (Figure 2A). Dicer is usually reported as a more powerful prognosticator for survival than Drosha [27, 28]. Stratified by ECOG, Dicer expression turned out to be significant for patients with normal performance status (ECOG = 0) only (Figure 2D). In general, patients with reduced performance status (ECOG = 1–4) have a more advanced disease and poor prognosis, independent of the tumor’s biological properties [33]. This might explain why Dicer expression had no impact on survival in this group of patients, and might also explain the differences of Dicer impact compared with other NSCLC studies, where ECOG performance is rarely mentioned. Our results clearly show that low Dicer expression is a significant negative prognostic marker in patients with normal performance status ECOG = 0 (Figure 2D). This patient group might therefore have an advantage in receiving adjunctive treatment. In addition, for patients with normal ECOG performance status, the low expression of Dicer was positively correlated with better survival rates in the patient group with squamous cell carcinoma (see Figure 2F), whereas Dicer expression had no prognostic impact on other histological subgroups (data not shown).

Dicer, Drosha, and miRNAs are involved in cell growth and differentiation, implying an impact on tumorigenesis [39]. Various studies focusing on Dicer and/or Drosha have confirmed this theory and revealed that these two regulators of the miRNA processing pathway play either a positive or a negative role in tumor transformation. There is evidence that reduced Dicer expression is associated with poor prognosis in NSCLC [28]. However, in vitro experiments showed that silencing of Dicer and Drosha decreases angiogenesis [24]. In our study, we found that increased expression of Dicer correlates with better prognosis. Neuroblastoma and leukemia are two other examples where low levels of Dicer and Drosha are significant predictive factors for poor outcomes [27, 40]. In other types of cancer, the importance of Dicer and Drosha might be the opposite. A recent study by Faber et al. using TMA technology and a scoring system like the one described here, found evidence that Dicer is a negative prognosticator for DSS in colorectal cancer [41]. Clearly, the functions of Dicer and Drosha are not fully understood in cancer development, and their functions appear to vary between different cancer types [42–44].

In most cancers, the majority of all miRNAs are downregulated, suggesting that most miRNAs have tumor suppressive effects [44]. Regulation of miRNA biogenesis is a complex process involving a myriad of different enzymes and proteins, where Dicer and Drosha are two key regulators necessary for the processing of most functional, mature miRNAs. We previously described the prognostic impact of miR-126 in NSCLC [19], where the co-expression of miR-126 and VEGF-A was a strong predictor for poor survival. We know that VEGF-A is a potent angiogenesis promoter, and miR-126 has been linked to angiogenesis in several other studies [11, 45, 46]. Interestingly, we found that the co-expression of Drosha and miR-126 also predicts poor survival, which is even more significant than the co-expression of miR-126 and VEGF-A reported previously [19]. Although not significant (p = 0.06), the combination of high Drosha and low miR-126 was the most favorable in relation to DSS (see Table 1), suggesting that Drosha in itself is not a good prognostic marker for overall survival in NSCLC, which is consistent with our univariate analyses (see Figure 3A). We tested the co-expressions of both Dicer and Drosha, in combination with the miRNAs miR-126 and miR-155 [47] in all 335 patients, and miR122a and let-7a in 40 randomly selected patients (data not shown). These tests demonstrated that the only combination with impact on DSS was the Drosha and miR-126 combination (see Figure 4). The significance of Drosha, as well as Dicer, in driving angiogenesis *in vitro* has been reported previously [24]. However, *in vivo* experiments showed that only Dicer reduces angiogenesis [48]. Our results imply that Drosha in itself is not a good prognostic marker in NSCLC, and that the effect of Drosha might be influenced by different miRNAs involved in tumor angiogenesis.

In addition, Dicer-independent, and probably Drosha-independent maturing of miRNAs is possible, suggesting alternative pathways and different roles for Dicer and Drosha in various cancers [49, 50]. As an example, miR-451 is processed by Ago, without the need for Dicer [51]. Several studies have also shown that knockdown of Dicer and Drosha only reduces a subset of miRNAs, implying alternative pathways for miRNA synthesis [49–51]. Further studies are clearly needed to investigate these possibilities.

## Conclusion

The immunohistochemical approach reveals the varying presence of Dicer and Drosha in NSCLC tumors, and these two enzymes may be important in NSCLC development. Our research points to Dicer as an important factor in regard to DSS in patients with normal ECOG, and implies that Drosha in combination with miR-126 and possibly other angiogenesis-related miRNAs, is a strong and important prognosticator for DSS in NSCLC. The Dicer and Drosha expression status in various histologic subtypes of lung cancer and at different stages of lung cancer development might explain abnormalities in miR profiles of NSCLC. Additional studies are needed, since optimized treatment of NSCLC requires better identification of high-risk patients who will benefit from adjuvant therapy.

## Electronic supplementary material

Additional file 1: Table S1: Prognostic Clinicopathologic Variables as Predictors for Disease-Specific Survival in 335 NSCLC Patients (Univariate Analyses; Log-rank Test) adapted from [19]. **Table S2.** Expression of Dicer, Drosha and miR-126 by histology. **Table S3.** Cox regression analysis summarizing significant independent prognostic factors in the total patient material with Dicer and Drosha as covariates on a continuous scale. **Table S4.** Expression of Dicer by histology, performance status, tumor differentiation, tumor status, nodal status and vascular infiltration. **Table S5.** Cox regression analysis summarizing significant independent prognostic factors exploring Dicer interaction with histology. **Table S6.** Cox regression analysis summarizing significant independent prognostic factors exploring Dicer interaction with ECOG. **Table S7.** Cox regression analysis summarizing significant independent prognostic factors exploring Dicer interaction with vascular infiltration. (PDF 343 KB)

Additional file 2: Figure S1: Normal lung tissue. **Figure S2.** Immunohistochemical (IHC) staining of Dicer in NSCLC tissues, representing (A) negative staining, (B) weak staining, (C) intermediate staining, and (D) strong staining. Dicer is found primarily in cytoplasm, see brown staining. **Figure S3.** Immunohistochemical (IHC) staining of Drosha in NSCLC tissues, representing (A) negative staining, (B) weak staining, (C) intermediate staining, and (D) strong staining. Drosha is primarily found in the nuclei, see brown staining. **Figure S4.** Correlation between Dicer and Drosha expression in the total patient material. **Figure S5.** Proportionality of the hazards. (PDF 703 KB)
