# Supplementary material for: Prognostic value of the MicroRNA regulators Dicer and Drosha in non-small-cell lung cancer: co-expression of Drosha and miR-126 predicts poor survival
Source: BMC Clin Pathol. 2014 Dec 11;14:45. doi: 10.1186/1472-6890-14-45 (PMC4269969; doi:10.1186/1472-6890-14-45)
Supplement: Supplementary file 1 — Additional file 1: Table S1: Prognostic Clinicopathologic Variables as Predictors for Disease-Specific Survival in 335 NSCLC Patients (Univariate Analyses; Log-rank Test) adapted from [19]. Table S2. Expression of Dicer, Drosha and miR-126 by histology. Table S3. Cox regression analysis summarizing significant independent prognostic factors in the total patient material with Dicer and Drosha as covariates on a continuous scale. Table S4. Expression of Dicer by histology, performance status, tumor differentiation, tumor status, nodal status and vascular infiltration. Table S5. Cox regression analysis summarizing significant independent prognostic factors exploring Dicer interaction with histology. Table S6. Cox regression analysis summarizing significant independent prognostic factors exploring Dicer interaction with ECOG. Table S7. Cox regression analysis summarizing significant independent prognostic factors exploring Dicer interaction with vascular infiltration. (PDF 343 KB) [file 12907_2014_190_MOESM1_ESM.pdf]

Table S1. Prognostic Clinicopathologic Variables as Predictors for Disease-Specific Survival in 335 NSCLC Patients (Univariate Analyses; Log-rank Test) adapted from [19].

Characteristic	Patients (n)	Patients (%)	Median survival (months)	5-Year survival (%)	P
**Age**					0.34
≤ 65 years	156	47	83	55	
> 65 years	179	53	NR	60	
**Sex**					0.20
Female	82	25	190	63	
Male	253	75	83	56	
**Smoking**					0.23
Never	15	5	19	43	
Current	215	64	NR	60	
Former	105	31	71	54	
**Performance status**					**0.013**
ECOG 0	197	59	NR	63	
ECOG 1	120	36	64	52	
ECOG 2	18	5	25	33	
**Weight loss**					0.71
< 10%	303	90	127	58	
> 10%	32	10	98	57	
**Histology**					**0.028**
SCC	191	57	NR	66	
Adenocarcinoma	95	28	54	45	
LCC	31	9	98	56	
BAC	18	5	NR	67	
**Differentiation**					**< 0.001**
Poor	138	41	47	47	
Moderate	144	43	190	64	
Well	53	16	NR	68	
**Surgical procedure**					**0.004**
Lobectomy + Wedge*	243	73	190	61	
Pneumonectomy	92	27	37	47	
**Pathological stage**					**< 0.001**
I	157	47	190	71	
II	136	41	61	51	
IIIa	42	12	17	23	
**Tumor status**					**< 0.001**
1	85	25	190	74	
2	188	56	84	57	
3	62	19	25	36	
**Nodal status**					**< 0.001**
0	232	69	190	66	
1	76	23	35	43	
2	27	8	18	18	
**Surgical margins**					0.29
Free	307	92	190	58	
Not free	28	8	47	47	
**Vascular infiltration**					**< 0.001**
No	284	85	190	58	
Yes	51	15	27	32	

Table S2. Expression of Dicer, Drosha and miR-126 by histology

Histology	Dicer*		Drosha**		miR-126***	
	**Mean**	**95% CI**	**Mean**	**95% CI**	**Mean**	**95% CI**
squam	1.28	1.16-1.40	1.47	1.36-1.58	1.27	1.14-1.41
adeno	0.95	0.78-1.12	1.28	1.13-1.43	0.92	0.75-1.10
bac	1.50	1.00-2.00	1.44	0.99-1.89	1.08	0.54-1.62
large-undiff	1.17	0.86-1.49	1.44	1.15-1.73	1.07	0.79-1.35
Total	1.18	1.09-1.28	1.41	1.33-1.49	1.14	1.05-1.24

* P = 0.008
** P = 0.282
*** P = 0.020

Table S3. Cox regression analysis summarizing significant independent prognostic factors in the total patient material with Dicer and Drosha as covariates on a continuous scale.

Factor	HR	95% CI	P
**Tumor status**			<0.001*
1	1.00		
2	1.84	1.10 –3.07	0.021
3	3.65	2.04 – 6.52	<0.001
**Nodal status**			<0.001*
0	1.00		
1	2.01	1.30 – 3.11	0.002
2	2.82	1.63 – 4.91	<0.001
**Histology**			0.006*
Squamous cell	1.00		
Adeno	1.85	1.24 – 2.76	0.003
Bac	0.51	0.06 – 4.08	0.527
Large undiff	0.81	0.41 – 1.59	0.536
**Differentiation**			0.016*
Poor	1.00		
Moderate	0.57	0.37 – 0.86	0.008
Well	0.55	0.27 – 1.09	0.085
**Performance status**			0.005*
Normal	1.00		
Slightly reduced	1.81	1.23 – 2.68	0.003
In bed < 50%	2.08	0.92 – 4.71	0.079
**Vascular infiltration**			
No	1.00		
Yes	2.25	1.42 – 3.58	0.001
**Dicer****	1.04	0.84 – 1.30	0.712
**Drosha*****	0.94	0.704 – 1.27	0.700

**Overall significance as a prognostic factor.*
***B=0.042*
****B=-0.058*
HR: Hazard ratio; CI: confidence interval

Table S4. Expression of Dicer by histology, performance status, tumor differentiation, tumor status, nodal status and vascular infiltration

	Dicer			
**Histology**	**Number**	**Mean**	**95% CI**	**P-value**
SCC	186	1.28	1.16-1.40	0.012
Other NSCLC	135	1.05	0.90-1.19	
**Performance status**				
ECOG = 0	191	1.18	1.06-1.30	0.915
ECOG = 1-2	130	1.19	1.05-1.33	
**Tumor differentiation**				
Poor	134	1.11	0.96-1.25	0.209
Moderate	142	1.28	1.14-1.42	
Well	45	1.12	0.88-1.37	
**Tumor status**				
1	81	1.36	1.18-1.55	0.072
2	180	1.11	0.99-1.23	
3	60	1.17	0.95-1.38	
**Nodal status**				
0	224	1.20	1.09-1.31	0.702
1	73	1.11	0.91-1.32	
2	24	1.24	0.91-1.58	
**Vascular infiltration**				
Yes	50	1.41	1.18-1.64	0.035
No	271	1.14	1.04-1.24	

Table S5. Cox regression analysis summarizing significant independent prognostic factors exploring Dicer interaction with histology.

Factor	HR	95% CI	P
**Tumor status**			<0.001*
1	1.00		
2	1.68	1.03 – 2.74	0.040
3	3.10	1.78 – 5.41	<0.001
**Nodal status**			<0.001*
0	1.00		
1	2.19	1.45 – 3.32	<0.001
2	3.04	1.77 – 5.23	<0.001
**Histology**			
SCC	1.00		
Other NSCLC	0.98	0.51 – 1.89	0.951
**Differentiation**			0.053*
Poor	1.00		
Moderate	0.66	0.45 – 0.97	0.034
Well	0.58	0.31 – 1.10	0.096
**Performance status**			
ECOG = 0	1.00		
ECOG = 1-2	1.80	1.26 – 2.59	0.001
**Vascular infiltration**			
No	1.00		
Yes	1.80	1.26 – 3.12	0.003
**Dicer****	0.87	0.68 – 1.10	0.257
**Dicer*histology*****	1.37	1.10 – 1.70	0.005

**Overall significance as a prognostic factor.*
***B = 0.313*
****B = -0.141*
HR: Hazard ration; CI: confidence interval

Table S6. Cox regression analysis summarizing significant independent prognostic factors exploring Dicer interaction with ECOG.

Factor	HR	95% CI	P
**Tumor status**			<0.001*
1	1.00		
2	1.58	0.97 – 2.58	0.067
3	2.96	1.70 – 5.15	<0.001
**Nodal status**			<0.001*
0	1.00		
1	2.21	1.45 – 3.35	<0.001
2	2.80	1.62 – 4.86	<0.001
**Histology**			
SCC	1.00		
Other NSCLC	1.57	1.07 – 2.30	0.022
**Differentiation**			0.092*
Poor	1.00		
Moderate	0.70	0.47 – 1.05	0.081
Well	0.58	0.31 – 1.09	0.090
**Performance status**			
ECOG = 0	1.00		
ECOG = 1-2	1.88	1.30 – 2.70	0.001
**Vascular infiltration**			
No	1.00		
Yes	2.02	1.28 – 3.18	0.003
**Dicer****	0.97	0.72 – 1.32	0.862
**Dicer*ECOG*****	1.12	0.83 – 1.50	0.475

**Overall significance as a prognostic factor.*
***B = -0.027*
****B = 0.629*
HR: Hazard ration; CI: confidence interval

Table S7. Cox regression analysis summarizing significant independent prognostic factors exploring Dicer interaction with vascular infiltration.

Factor	HR	95% CI	P
**Tumor status**			<0.001*
1	1.00		
2	1.58	0.97 – 2.58	0.067
3	2.96	1.70 – 5.15	<0.001
**Nodal status**			<0.001*
0	1.00		
1	2.21	1.45 – 3.35	<0.001
2	2.80	1.62 – 4.86	<0.001
**Histology**			
SCC	1.00		
Other NSCLC	1.57	1.07 – 2.30	0.022
**Differentiation**			0.092*
Poor	1.00		
Moderate	0.70	0.47 – 1.05	0.081
Well	0.58	0.31 – 1.10	0.090
**Performance status**			
ECOG = 0	1.00		
ECOG = 1-2	1.88	1.30 – 2.70	0.001
**Vascular infiltration**			
No	1.00		
Yes	2.02	1.28 – 3.18	0.003
**Dicer****	1.04	0.84 – 1.29	0.708
**Dicer*vascular infiltration*****	0.45	0.48 – 1.49	0.563

**Overall significance as a prognostic factor.*
***B = 0.041*
****B = -0.166*
HR: Hazard ration; CI: confidence interval
