# Supplementary material for: Prognostic value of the MicroRNA regulators Dicer and Drosha in non-small-cell lung cancer: co-expression of Drosha and miR-126 predicts poor survival
Source: BMC Clin Pathol. 2014 Dec 11;14:45. doi: 10.1186/1472-6890-14-45 (PMC4269969; doi:10.1186/1472-6890-14-45)

Figure 1S: Normal lung tissue

A: Dicer

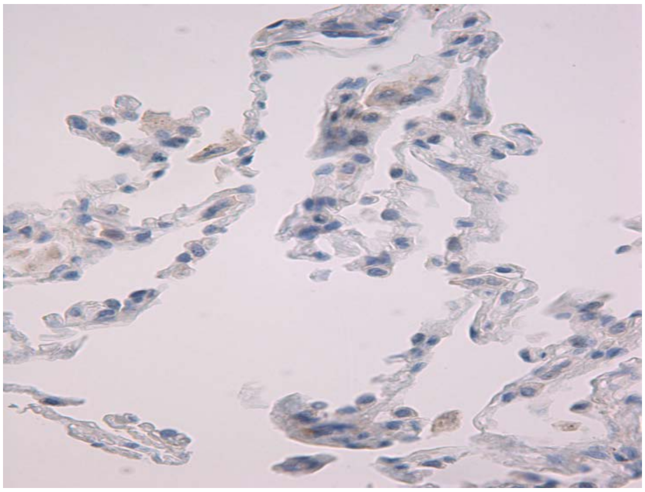

B: Drosha

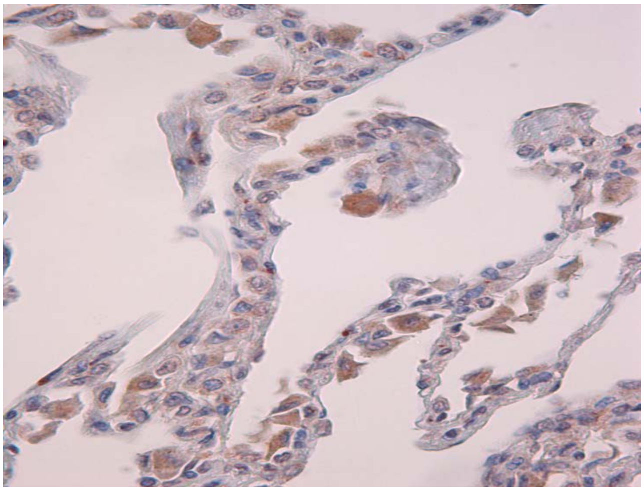

Figure S2 Immunohistochemical (IHC) staining of Dicer in NSCLC tissues, representing (A) negative staining, (B) weak staining, (C) intermediate staining, and (D) strong staining. Dicer is found primarily in cytoplasm, see brown staining.

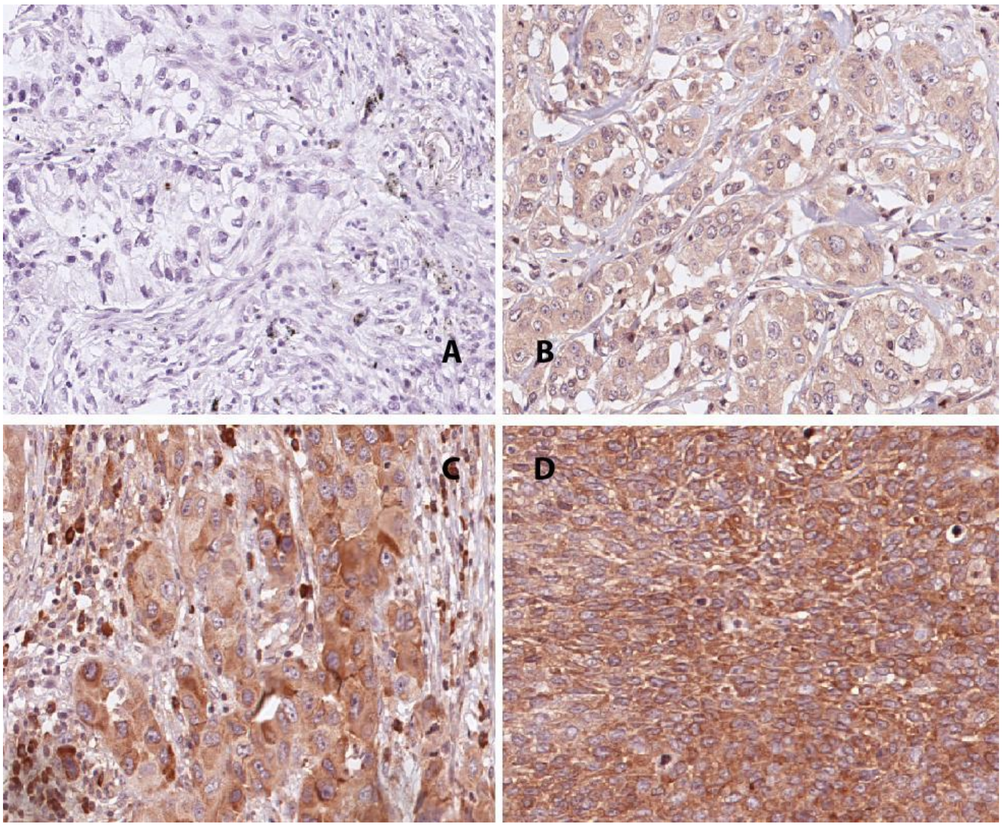

Figure S3 Immunohistochemical (IHC) staining of Drosha in NSCLC tissues, representing (A) negative staining, (B) weak staining, (C) intermediate staining, and (D) strong staining. Drosha is primarily found in the nuclei, see brown staining.

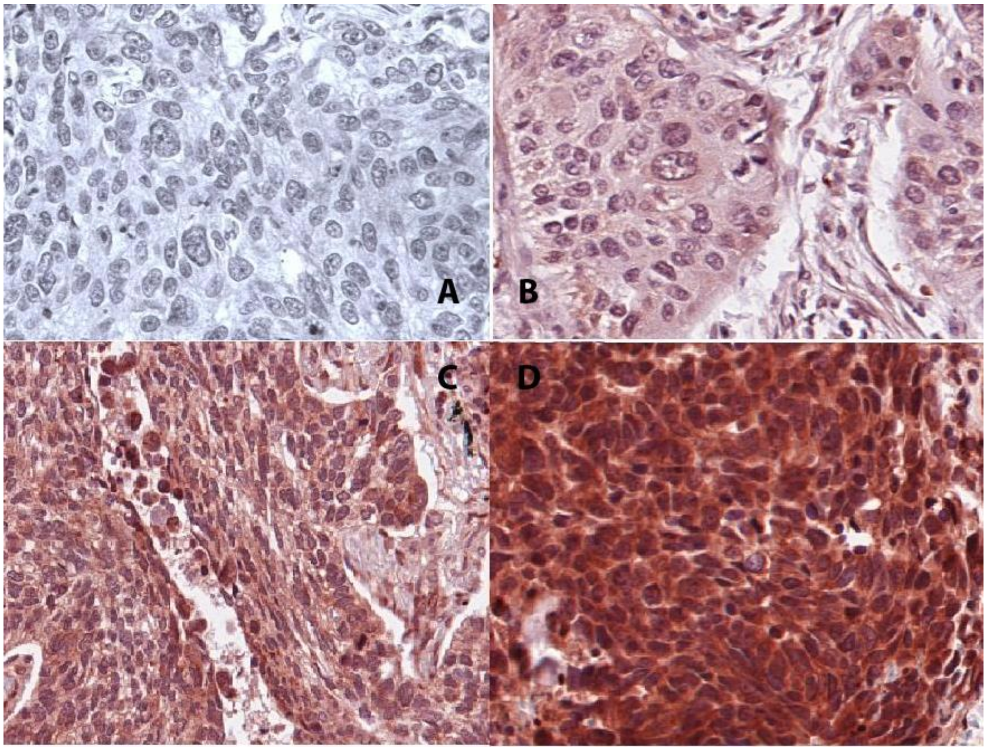

Figure S4 Correlation between Dicer and Drosha expression in the total patient material.

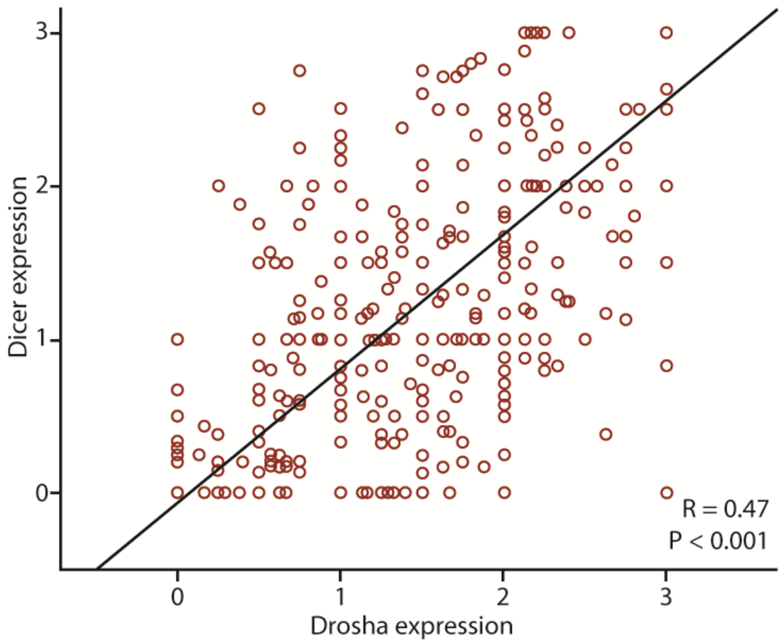

Figure S5: Proportionality of the hazards

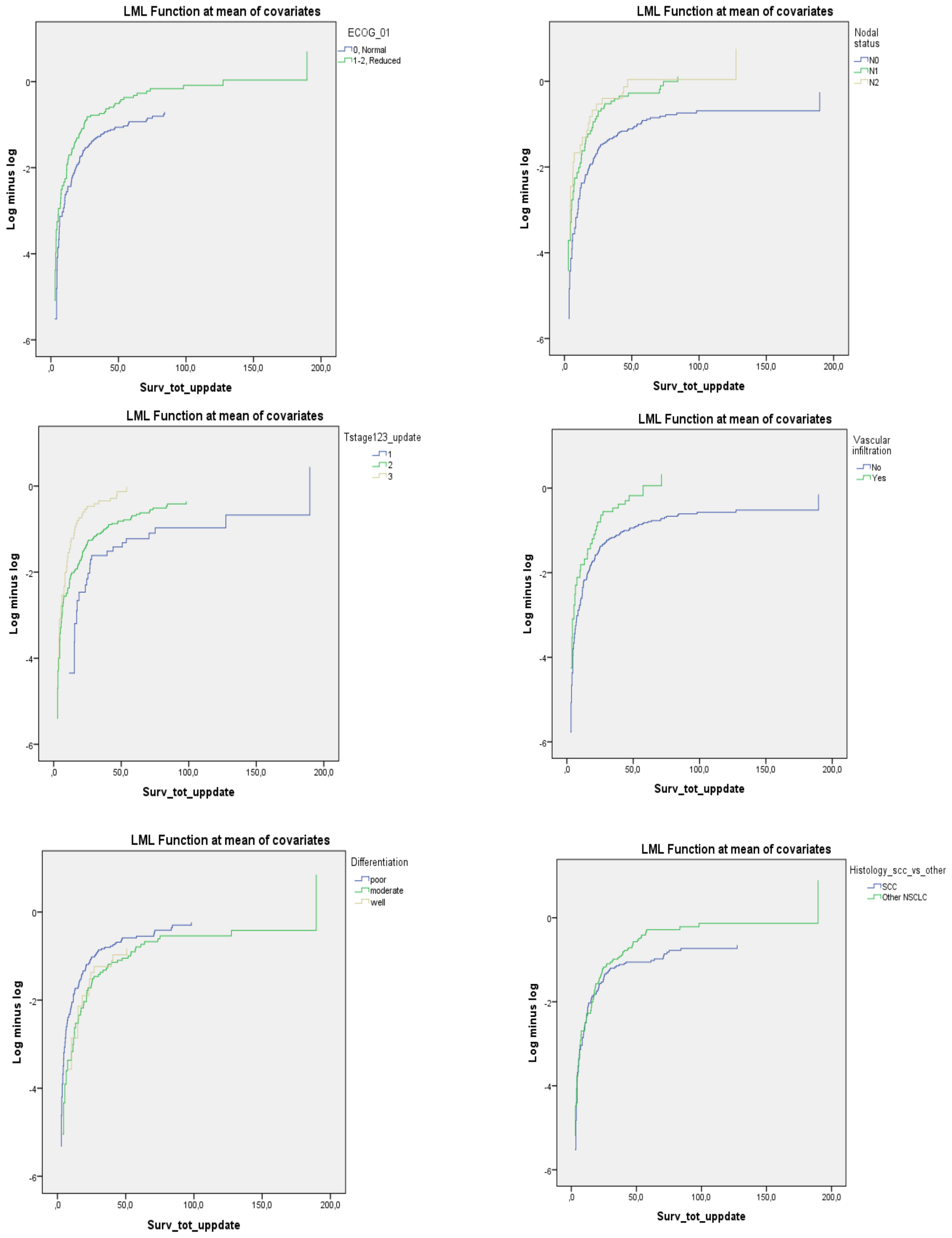

Supplement: Supplementary file 2 — Additional file 2: Figure S1: Normal lung tissue. Figure S2. Immunohistochemical (IHC) staining of Dicer in NSCLC tissues, representing (A) negative staining, (B) weak staining, (C) intermediate staining, and (D) strong staining. Dicer is found primarily in cytoplasm, see brown staining. Figure S3. Immunohistochemical (IHC) staining of Drosha in NSCLC tissues, representing (A) negative staining, (B) weak staining, (C) intermediate staining, and (D) strong staining. Drosha is primarily found in the nuclei, see brown staining. Figure S4. Correlation between Dicer and Drosha expression in the total patient material. Figure S5. Proportionality of the hazards. (PDF 703 KB) [file 12907_2014_190_MOESM2_ESM.pdf]
